# P62 The benefits and limitations of telemedicine appointments for assessing children and young people with rheumatic conditions in Canada: A patient-led survey

**DOI:** 10.1093/rap/rkac067.062

**Published:** 2022-09-28

**Authors:** Jennifer Wilson, Wendy Costello, Saskya Angevare, Richard Beesley

**Affiliations:** Cassie And Friends Society, Vancouver, Canada; European Network for Children with Arthritis, Geneva, Switzerland; iCAN Ireland, Bansha, Ireland; KAISZ, Amsterdam, Netherlands; European Network for Children with Arthritis, Geneva, Switzerland; Juvenile Arthritis Research, Tonbridge, United Kingdom; European Network for Children with Arthritis, Geneva, Switzerland

## Abstract

**Introduction/Background:**

During the COVID-19 (coronavirus) pandemic, some healthcare provision shifted to remote, technology-assisted appointments (telemedicine). This study sought the views of parents/carers about telemedicine, identifying the benefits and limitations, to assist in improvement to future service provision.

**Description/Method:**

An online survey was developed and shared via social media and direct contacts, targeted at parents of children with rheumatic and autoinflammatory conditions in Canada. Fieldwork took place during May 2021. Consent was provided during enrolment.

**Discussion/Results:**

A total of 157 responses were received (78% female, median age 12). The primary diagnosis for the majority was Juvenile Idiopathic Arthritis (JIA; 39% polyarticular, 15% oligoarticular, 8% enthesitis-related JIA, 6% psoriatic, and 9% systemic). Respondents reported in-person appointments represent a considerable time burden (87% travel more than an hour to attend; 40% take a full day (or more) out of school to attend; 38% of parents take a full day off work). During the pandemic, the proportion having a telemedicine appointment increased from 5% to 82%. Table 1 shows the scores (1 worst, 5 best) given by parents about their telemedicine experience. Overall, most aspects scored positively (p<.05). However, parents felt telemedicine was not as good as in-person appointments (mean 2.66, 95% CI 2.42-2.90).

Overall 61% said they would prefer the next appointment to be in-person, while 31% were amenable to some combination of in-person and virtual care.

**Key learning points/Conclusion:**

There are advantages to telemedicine, notably saving time and making appointments accessible, and overall parents reported satisfaction with remote appointments. However, parents continue to report the value of in-person appointments.

**P62 Table 1:** Mean scores for a range of aspects of telemedicine (1-worst; 5-best).

**Aspect**	**Mean (95% CI)**
Easy to schedule	4.33 (4.14, 4.52) *
On time	4.07 (3.85, 4.28) *
Enough time with doctor	4.24 (4.02, 4.45) *
As good as in-person visit	2.66 (2.42, 2.90) **
Easier to see doctor	3.51 (3.25, 3.77) *
Easy to sign-in	4.25 (4.06, 4.43) *
Quality of video	3.87 (3.66, 4.07) *
Quality of sound	3.94 (3.75, 4.14) *
Able to speak freely	4.05 (3.85, 4.24) *
Able to understand doctor	4.09 (3.90, 4.28) *
Quality of care provided	3.78 (3.56, 4.00) *
Overall telemedicine experience	3.78 (3.57, 3.99) *

* Positive score (p<.05)

** Negative score (p<.05).

